# Globular Head-Displayed Conserved Influenza H1 Hemagglutinin Stalk Epitopes Confer Protection against Heterologous H1N1 Virus

**DOI:** 10.1371/journal.pone.0153579

**Published:** 2016-04-18

**Authors:** Miriam Klausberger, Rupert Tscheliessnig, Silke Neff, Raffael Nachbagauer, Teddy John Wohlbold, Monika Wilde, Dieter Palmberger, Florian Krammer, Alois Jungbauer, Reingard Grabherr

**Affiliations:** 1 Department of Biotechnology, University of Natural Resources and Life Sciences Vienna, Vienna, Austria; 2 Austrian Centre of Industrial Biotechnology, Vienna, Austria; 3 Faculty of Life Sciences, University of Vienna, Vienna, Austria; 4 Department of Microbiology, Icahn School of Medicine at Mount Sinai, New York, NY, United States of America; 5 Graduate School of Biomedical Sciences, Icahn School of Medicine at Mount Sinai, New York, NY, United States of America; Monash University, Australia, AUSTRALIA

## Abstract

Significant genetic variability in the head region of the influenza A hemagglutinin, the main target of current vaccines, makes it challenging to develop a long-lived seasonal influenza prophylaxis. Vaccines based on the conserved hemagglutinin stalk domain might provide broader cross-reactive immunity. However, this region of the hemagglutinin is immunosubdominant to the head region. Peptide-based vaccines have gained much interest as they allow the immune system to focus on relevant but less immunogenic epitopes. We developed a novel influenza A hemagglutinin-based display platform for H1 hemagglutinin stalk peptides that we identified in an epitope mapping assay using human immune sera and synthetic HA peptides. Flow cytometry and competition assays suggest that the identified stalk sequences do not recapitulate the epitopes of already described broadly neutralizing stalk antibodies. Vaccine constructs displaying 25-mer stalk sequences provided up to 75% protection from lethal heterologous virus challenge in BALB/c mice and induced antibody responses against the H1 hemagglutinin. The developed platform based on a vaccine antigen has the potential to be either used as stand-alone or as prime-vaccine in combination with conventional seasonal or pandemic vaccines for the amplification of stalk-based cross-reactive immunity in humans or as platform to evaluate the relevance of viral peptides/epitopes for protection against influenza virus infection.

## Introduction

Immunization is—at present—the only effective prophylaxis against seasonal influenza virus infections in humans. Current vaccination strategies aim at the induction of neutralizing antibodies against the immunodominant hemagglutinin (HA) globular head domain. These antibodies inhibit receptor binding of the virus and are presently the only correlate of protection against infection. Rapid antigenic evolution of the HA head, however, renders the conferred immunity strain-specific and may not be effective against drifted strains that emerge in the upcoming influenza season [1]. In contrast, eliciting an immune response to epitopes that are highly conserved among subtypes, such as those located in the HA stalk domain, has been shown to result in broader cross-protective immunity [2]. Therefore, a vaccine that focuses the immune response to neutralizing epitopes in the stalk domain could be key in providing cross-reactive immunity against drifted influenza virus strains.

In this study, we tested a novel display-format by utilizing a soluble insect-cell expressed influenza A H3 HA as carrier for the display of stalk sequences from a heterosubtypic H1 HA. Putative epitopes were identified in an epitope mapping assay with human sera that originated from a clinical trial with a new generation of replication-deficient intranasal monovalent seasonal delNS1 vaccine based on A/New Caledonia/20/1999 (H1N1) [3]. Sera were evaluated for their reactivity with short synthetic peptides spanning the entire HA sequence of the vaccine strain. Using binding data along with computer-aided calculations of spatial proximities between peptides in the HA 3D structure, putative linear and discontinuous stalk epitopes were identified. Putative stalk epitopes have proven not to recapitulate the epitopes of previously isolated broadly neutralizing antibodies (bnAbs) that bind to the HA stalk including mAbs 6F12 [4] and KB2 [5]. By virtue of the immunodominance of the head domain, such bnAbs are only occasionally found in humans exposed to seasonal influenza viruses by infection or vaccination [6–11] and only amount for levels up to 100,000-fold lower than head-directed antibodies [10]. We aimed to enhance immunogenicity of selected stalk epitopes by engineering them into the immunodominant antigenic site B of an H3 HA head, which serves to display such epitopes on a protein-based carrier. The established protein-based epitope carrier allowed us to evaluate the protective efficacy of identified stalk sequences. Vaccination with 25-mer peptides displayed using this system provided up to 75% protection from mortality upon lethal infection of mice with a heterologous H1 challenge virus and elicited serum antibodies that bind to the parental HA antigen.

## Materials and Methods

### Synthesis of peptide membranes

A technique originally developed by Frank [12] and modified by Pflegerl and colleagues [13] was used for synthesis of peptide arrays with the MultiPep Multiple Peptide Synthesizer (Intavis Bioanalytical Instruments AG, Cologne, DE). Peptides were C-terminally immobilized on cellulose sheets via di-ß-alanin anchors and 112 decapeptides (peptide sequences are provided in S1 Table) covering the entire HA sequence from influenza virus A/New Caledonia/20/1999 (NC99, H1N1) were synthesized using the conventional Fmoc technique. Peptides were designed to have five amino acid (aa) sequence overlaps. After the coupling reaction, all N—termini were acetylated and side chain protection groups were cleaved. Each peptide membrane included negative control peptide spots (deca-alanin) and positive control peptide spots (WSHPQFEK) that bind streptavidin.

### Preparation of human serum samples

Human serum samples were obtained from a clinical trial (Clinical.Trials.gov Identifier: NCT00724997) with healthy volunteers that received either a novel type of seasonal monovalent live-attenuated influenza virus vaccine (LAIV; delNS1-H1N1) based on NC99 or placebo [3]. A total of sixteen aliquots of pre-immune and immune serum samples were obtained from twelve volunteers that had received the highest vaccine dose (7.7 log^10^ median tissue culture infective dose, TCID^50^) and four placebo controls (receiving stabilizing buffer only). The protocol was approved by the ethics committee at the Department of Clinical Pharmacology, Medical University of Vienna, Austria, and was conducted in compliance with good clinical practice guidelines and the Declaration of Helsinki. Study participants provided written consent using approved forms and procedures.

Frozen samples were thawed at 4°C, centrifuged, sterile-filtered and serum IgGs from individual donors were purified using Protein G Fast Flow Sepharose as affinity chromatography support and a FPLC pump system with a UV-detector, both from GE Health Care Life Sciences, Little Chalfont, UK. Before and after loading the serum samples, phosphate buffered saline (PBS) was applied to equilibrate the column and wash-off unbound proteins, respectively. Serum IgGs were eluted using 0.2 M acetic acid with 20% (v/v) ethylenglycol and fractions were collected by monitoring the absorbance at 280 nm. Eluates of purified IgGs were subsequently neutralized with 1 M sodium carbonate and labelled with a 20-fold molar excess of 20 mM biotinamidohexanoic acid N-hydroxysuccinimide ester in dimethylformamide. After one hour of incubation, biotinylated IgGs were dialyzed against PBS supplemented with 0.1% (w/v) sodium azide. The samples were stored at 4°C until further use.

### Binding assays with purified human serum samples on peptide membranes

A chemiluminescence binding assay was employed to identify antigenic regions in the influenza hemagglutinin. Blank assays (without serum incubation) served to establish assay conditions that yield least unspecific binding of the streptavidin conjugate. Then, different concentrations of serum IgGs (5–50 μg/mL), different incubation times with serum IgG (15–60 minutes) and the addition of urea (0.3–4 M) to the serum IgG incubation and washing buffers were tested to keep unspecific IgG binding low. The optimized assay protocol was as follows: Peptide membranes were re-equilibrated for 30 minutes in 20% (v/v) methanol and then washed with PBS containing 0.1% (v/v) Tween 20 (PBS-T). Membranes were blocked with PBS-T containing 3% (w/v) bovine serum albumin (BSA) for two hours, washed again with PBS-T and then incubated for one hour with 50 μg/mL biotin-labeled serum IgGs diluted in incubation buffer (PBS-T with 1% (w/v) BSA and 1.2 M urea). After a wash with PBS-T containing 1.2 M urea, membranes were incubated with a streptavidin-horseradish peroxidase (HRP) conjugate (GE Healthcare Life Sciences, Little Chalfont, UK) at a 1:3000 dilution in PBS-T containing 0.8 M NaCl and 1% (w/v) BSA for one hour. Membranes were finally washed with PBS-T supplemented with 0.8 M NaCl, incubated for 5 minutes with a Super Signal Chemiluminescent Substrate (Thermo Fisher Scientific, Waltham, MA). Chemiluminescence of the peptide spots was measured on a Lumi-Imager^™^ Workstation (F.Hoffmann-La Roche AG, Basel, CH). Image spots were normalized and negative (deca-alanin) and positive control (Strep-tag) peptide spots were defined as 0% and 100% intensity respectively. Signal intensities (%) of serum IgGs binding to the individual hemagglutinin peptide spots were calculated.

### Prediction of potential discontinuous epitopes

For the identification of potential discontinuous epitopes we computed data on the spatial proximity of the individual peptides in the HA 3D structure. The sequence of A/New Caledonia/20/1999 was mapped onto the crystallographic structure of the hemagglutinin precursor HA0 of A/Puerto Rico/8/1934 (PR8) (PDB#: 1RU7). We scanned the homologous model for amino acid sequences that matched at least 50% of the sequences of the stalk peptides and identified 12 peptides: 66–68, 86–89, 69, 70, 73, 77, 87, 88, 89, 96, 98, 100. We computed average relative distances of the centre of masses of the amino acids of a peptide to those of complement peptides. As each peptide is a 3D object in space, we also computed the relative vectors from the individual centre of masses of the amino acids. Vectors were normalized and averaged over all vectors to determine the particular orientation of the peptides. Consequently, we computed the average spatial distances of each peptide to its pendants and analyzed their orientation in respect to each other. Relative distances and orientations were obtained by algorithms written in Mathematica (http://www.wolfram.com/mathematica/?source=nav) and the 3D structure was plotted with Visual Molecular Dynamics (VMD) (http://www.ks.uiuc.edu/Research/vmd/). Based on the mapped HA structure, putative epitopes were compared to the footprint of crystallized broadly-neutralizing stalk monoclonal antibodies (mAbs) C179 [14], 39.29 [15] and CR9114 [16] (S3 Fig).

### Insect cells

*Sf9* insect cells (ATCC # CRL-1711) were routinely propagated in TNM-FH medium (Gemini Bio-Products, West Sacramento, CA) supplemented with 0.1% (v/v) Pluronic 68 (Sigma, St. Louis, MO), 10% (v/v) fetal bovine serum (FBS) (Atlanta Biologicals, Norcross, GA) and a Penicillin-Streptomycin antibiotic mixture (Life Technologies, Carlsbad, CA) at 27°C. Baculovirus amplification was performed in the presence of 3% (v/v) FBS. BTI-TN-5B1-4 (High Five—Vienna Institute of Biotechnology subclone) [17] cells were used for the expression of soluble influenza A hemagglutinin-based antigens and maintained at 27°C in HyClone SFX serum free media (Fisher Scientific, Hampton, NH) supplemented with Penicillin-Streptomycin antibiotic mixture.

### Viruses

Pandemic virus A/Netherlands/602/2009 (NL09, pH1N1) was propagated in 8- to 10-day-old embryonated chicken eggs for 48 h at 37°C and titered on Madin-Darby canine kidney (MDCK) cells in the presence of tosyl phenylalanyl chloromethyl ketone (TPCK)-treated trypsin.

### Generation of DNA and protein vaccine antigens

The HA nucleic acid sequences of A/New Caledonia/20/1999 (NC99, H1N1, GenBank: CY031336.1) and A/Hiroshima/52/2005 (HIR05, H3N2, GenBank: EU283414.1) were derived as described elsewhere [17]. Selected H1 subtype stalk peptides of about 25 aa in size (peptide 66–69: AKLRMVTGLRNIPSIQSRGLFGAIA, 86–89: KVDDGFLDIWTYNAELLVLLENERT, 106–109: *S*-VYQILAIYSTVASSLVLLVSLGAIS, 69+73: QSRGLFGAIA-GGGGG-VDGWYGYHHQ and 73+96: *S*-VDGWYGYHHQ-GGGGG-IGNGCFEFYH) were inserted into the antigenic site B loop in the globular head domain of an H3 subtype influenza HA (HIR05, phylogenetic group 2) after L173 (H3 numbering including signal peptide) using overlap-extension PCR. Putative discontinuous epitopes were separated by a 5x-glycine linker and a charged serine residue was introduced before hydrophobic valine residues to improve peptide display (as for peptide 73 and 106) [18]. Modified HA genes and *wild type* negative control H3 HA (HIR05) genes were cloned into a modified pFastBac vector (Invitrogen, Carlsbad, CA) under the control of the baculovirus polyhedrin promoter using *BamHI* and *NotI* restriction endonucleases (New England Biolabs, Ipswich, MA). The inserts were designed to yield soluble HA proteins with a thrombin cleavage site, a T4 foldon trimerisation domain and a C-terminal hexahistidine-tag for the generation of soluble HA protein antigens as described in [19]. Soluble HA from pandemic virus A/California/7/2009 (CAL09) and a chimeric cH6/1 (containing an H6 head on an H1 stalk) contained a GCN4pII trimerisation domain and a C-terminal Strep-Tag II sequence to prevent background signals in serological assays as described in [20]. Modified full-length HA proteins (including transmembrane domain and cytoplasmic tail) that display selected stalk peptides were cloned into a pFastBac Dual vector (Invitrogen, Carlsbad, CA) to yield insect cell surface-expressed proteins for flow cytometry analysis. Recombinant bacmids for the expression of soluble and cell-surface expressed HA proteins were generated using the Bac-to-Bac System and *E*.*coli* DH10Bac and were isolated using a PureLink Plasmid Filter Midiprep Kit, all from Invitrogen, Carlsbad, CA. Recombinant baculoviruses were generated using Cellfectin II transfection reagent (Invitrogen, Carlsbad, CA) and were rescued from *Sf9* cells and amplified to a passage 3 virus stock. High Five cells were infected with the recombinant baculoviruses at a multiplicity of infection of approximately 10 and cells were cultured at 28°C shaking. Recombinantly expressed soluble HAs were purified using a Nickel-nitrilotriacetic acid resin (Qiagen, Venlo, VL) as described in [20]. Protein concentration was quantified using a Quickstart Bradford Dye Reagent (Bio-Rad Laboratories, Inc., Hercules, CA) with a BSA standard curve. Protein purity, integrity and identity were assessed by sodium dodecyl sulfate polyacrylamide gel electrophoresis (SDS-PAGE) (4–20% polyacrylamide—Mini PROTEAN TGX gels, Bio-Rad Laboratories, Inc. Hercules, CA), Coomassie staining and Western blots and were performed as described in [21] using pan-H3 HA-specific mAb 12D1 [22] (S4 Fig). Full-length sequences of the respective constructs were cloned into a modified pCAGGS mammalian expression vector under control of the chicken beta-actin promoter using *SacI* restriction endonuclease (New England Biolabs, Ipswich, MA) and were utilized as DNA vaccine. Plasmid DNA was isolated using the NucleoBond Xtra Endotoxin-free (EF) Maxiprep Kit (Macherey-Nagel GmbH & Co KG, Düren, DE) and DNA concentration was measured using a NanoDrop Spectrophotometer (Thermo Fisher Scientific, Waltham, MA).

### Flow cytometry

To evaluate whether displayed putative H1 HA stalk epitopes recapitulate the epitopes of bnAbs that bind the HA stalk, epitope display vectors were expressed as full-length HA proteins on the surface of High Five insect cells. Cells were stained with 100 μL of pan-H1 mAb 6F12 [4], group 1-specific mAbs 3N6 [23] and KB2 [5] and pan-H3 mAb 12D1 [22] as expression control at a concentration of 5 μg/mL, followed by either an anti-mouse (6F12, 12D1, KB2) or anti-human (3N6) Alexa Fluor 488-labelled secondary antibody (Life Technologies, Carlsbad, CA) at a dilution of 1:1000 and analysed using a FACSCalibur flow cytometer (BD Biosciences, San Jose, CA) and the FlowJo software (TreeStar, Ashland, OR).

### Mouse immunization and challenge

Animal experiments were performed in female 6 to 8 week-old BALB/c mice (Jackson Laboratories, Bar Harbor, ME) according to a protocol approved by the Icahn School of Medicine at Mount Sinai Institutional Animal Care and Use Committee (permit LA12-00028). Animals were kept on a 12-hour light/dark cycle and had free access to food and water. Mice were anesthetized by intraperitoneal (i.p.) injection of 0.1 mL of a ketamine/xylazine mixture (0.15 mg/kg and 0.03 mg/kg) before intranasal or electroporation procedures. The four study groups (N = 5 each) received a DNA prime with 40 μg pCAGGS plasmid in water encoding full-length stalk peptide carrier HAs (abbreviated HIR05/NC99-Ep66-69, HIR05/NC99-Ep86-89, HIR05/NC99-Ep69+73 or HIR05/NC99-Ep73+96) in the left calf muscle by *in vivo* electroporation using a TriGrid delivery system (Ichor Medical Systems, San Diego, CA). Three and six weeks later (day 21 and 42) animals were boosted by simultaneous intranasal (i.n.) and intramuscular (i.m.) immunization with 2.5 μg of the respective purified soluble stalk peptide-carrier HA protein in PBS adjuvanted with 2.5 μg poly(I.C) (Invitrogen, Carlsbad, CA). The negative control group (N = 5) received a DNA prime encoding full-length *wild type* HIR05 HA and two adjuvanted protein boosts (2.5 μg) with soluble *wild type* HIR05 HA. The positive 'standard of care' control group (N = 5) received a single immunization with an unadjuvanted aliquot of the trivalent inactivated influenza vaccine (TIV) Agriflu^®^ (Novartis AG, Basel, CH) intramuscularly on day 42. The aliquot corresponded to 1 μg HA per vaccine strain and was from the 2010/11 influenza season (the H1 HA is from pandemic CAL09). Three weeks after the last immunization (day 63) blood was drawn from anesthetized mice by submandibular bleeding for immunological assays. Immunized mice were then challenged with 5 mLD_50_ of the heterologous pH1N1 strain A/Netherlands/602/2009 (NL09). One apparently healthy mouse in the study group HIR05/NC99-Ep66-69 died prior to the challenge experiment due to an undetermined reason and was precluded from further calculations. Animals were closely observed for signs of discomfort. A body condition scoring (BCS) chart was used as an endpoint criterion. The body condition scoring is on a scale of 1 to 5. According to this system, mice with a BCS of 1–2 are emaciated or under-conditioned, respectively. Mice with a BCS of 4–5 are over-conditioned and obese, respectively. A BCS score of 3 is the desirable score, where the animal is well conditioned. If the animal obtained a BCS score equal to or less than 2, the animal was humanely euthanized via CO_2_ inhalation following Mount Sinai Centre for Comparative Medicine and Surgery guidelines. Above that, mice were monitored daily for signs of distress and sick or lethargic animals were reported for immediate evaluation by a veterinarian. Infected animals were also closely observed for other symptoms such as labored breathing, lethargy, hair loss, anorexia and any behavioural changes. Furthermore, post-infection body weight was monitored daily for 14 days and if a mouse was found to lose greater than 20% of its pre-infection weight, it was humanely euthanized by CO_2_ inhalation following Mount Sinai Center for Comparative Medicine and Surgery guidelines.

### Serological assays

A quantitative ELISA served to assess whether mice mounted serum antibodies that are able to bind to the native HA antigen. Individual sera were assayed for titers specific for the rHA of pandemic CAL09 with a GCN4pII trimerisation domain and a C-terminal Strep-Tag II as described in [20].

To investigate the presence of stalk-reactive antibodies in the sera of vaccinated mice we performed a competitive ELISA and probed post-vaccination serum samples for the presence of KB2-like antibodies. Immulon 4HBX^®^ultra-high-binding polystyrene plates (Thermo Fisher Scientific, Waltham, MA) were coated with chimeric cH6/1 HA (carrying the head domain of an H6 HA on the stalk of an H1 HA and a GCN4pII trimerisation domain and C-terminal Strep-Tag II) [20] at a concentration of 2 μg/mL in coating buffer (0.1 M Na_2_CO_3_/NaHCO_3_, pH 9.2, 50 μl/well) at 4°C overnight. Plates were blocked with PBS-T and 3% (w/v) non-fat dry milk and sample wells were then incubated with 2-fold dilutions of individual serum samples (100 μl per well in PBS-T with 1% non-fat dry milk (starting concentration: 1:50 dilution) for 2 hours. Plates were washed and incubated with 100 μL of 10 μg/mL biotinylated mAb KB2 [5] for 1 hour. After washing, plates were incubated with horseradish peroxidise-conjugated streptavidin (Merck Millipore, Billerica, MA) at a dilution of 1:5000 for 1 hour, unbound streptavidin was removed and the colorimetric change was measured as the optical density (O.D. 490 nm) on a Synergy 4 (BioTek, Winooski, VT) microplate reader. The percentage of competition was calculated as the reduction in mean absorbance when serum IgG binding competition occurred.

### Statistical Analysis

Statistical significance was analysed using GraphPad Prism (GraphPad Software, Inc., La Jolla, CA). A P value of <0.05 was considered statistically significant. Group comparisons were performed with a one-way analysis of variance (ANOVA) with a Turkey post-test. Kaplan-Meier Survival Curves were analysed with the Log-Rank Test.

## Results

### Human immune serum antibodies bind to synthetic HA stalk peptides

**Fig 1:**
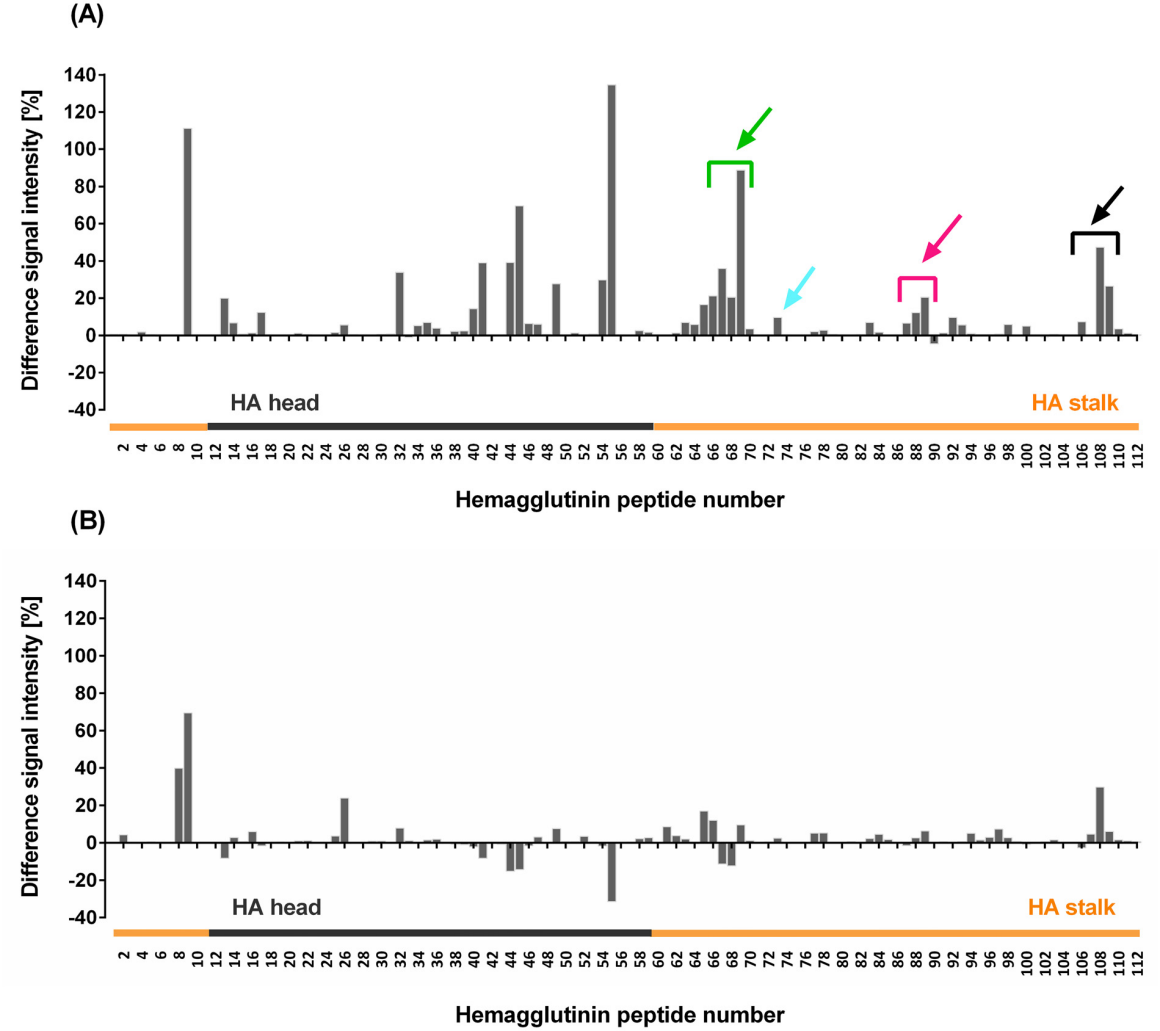
Human immune serum IgGs bind to synthetic HA stalk peptides. Human pre-immune and immune sera from individuals receiving a monovalent live-attenuated A (H1N1) virus vaccine based on NC99 (A) and placebo controls (B) were used in an epitope mapping experiment. Individual sera were evaluated for binding to 112 decapeptides (having overlaps of 5 aa) representing the entire NC99 HA sequence in a chemiluminescence binding assay. Image spots were normalized and negative (deca-alanin) and positive control (Strep-tag) peptide spots were defined as 0% and 100% intensity respectively. Signal intensities (%) of serum IgGs binding to the individual hemagglutinin peptide spots were calculated. Bars represent mean differences in peptide-specific chemiluminescence signal intensities between immune and pre-immune sera from twelve vaccinated individuals (Fig 1A) and four placebo controls (Fig 1B). Arrows indicate interesting antigenic peptide stretches and individual peptides of the stalk domain and include peptide Nos. 64–69 (green), 73 (cyan), 87–89 (pink) and 106–109 (black). HA stalk peptides are highlighted in orange. The two HA domains are roughly demarcated by peptides no. 11 and 58, as they harbour the two conserved cystine residues that act as boundary between them [20].

To identify antigenic regions in the influenza HA protein, we performed a chemiluminescence binding assay with purified biotinylated human sera from donors participating in a clinical trial (Clinical.Trials.gov Identifier: NCT00724997) and 112 immobilized decapeptides spanning the entire HA sequence of the monovalent seasonal NC99 (sH1N1) vaccine strain [3]. Calculation of mean differences in peptide-specific chemiluminescence signal intensities between pre- and post-vaccination sera from twelve vaccinated individuals suggest that vaccination with the LAIV induced antibodies that are able to bind to several synthetic peptides located in the HA head and stalk region (Fig 1A). The greatest mean increase in peptide-specific serum-reactivity (Fig 1A) and greatest diversity among individual sera (S1A Fig) was observed for HA stalk peptide 9 (mean: +111%; range: (-58)–564) and HA head peptide 55 (mean: +135%; range: (-39)–704) (see S1 Table for peptide sequences). For several peptides we also observed considerable sero-reactivity (Fig 1B) and highly diverse individual serological profiles with control sera (S1B Fig). The fluctuation in mean responses with control sera, however, seemed to be largely driven by a single donor (S1B Fig). To investigate whether the magnitude of head-specific HI response post vaccination correlates with the binding profile to lead head and stalk peptides, we clustered the study group into non, medium or high HI-responders (no, 2-4-fold or 16-fold-increase of HI activity in post-vaccination sera respectively). Non and high-HI responders showed rather consistent weak peptide-specific responses with all peptides, apart from stalk peptide 9. Medium-responders were shown to be largely responsible for the wide-ranging binding spectrum and for highest responses with head and stalk peptides (S2 Fig). As our focus were antigenic peptides from the HA stalk, we graphically identified and selected stalk peptides with highest mean increases in sero-reactivity and little sero-reactivity with control sera. As stalk peptide 9 showed the highest mean increase in sero-reactivity with control sera (+70%, Fig 1B) it was omitted from further experiments. Three separate stretches of two to five contiguous decapeptides in the HA stalk (peptide Nos. 65–69, 87–89 and 106–109, excluding peptide No. 107) exhibited considerable increases in mean serum-reactivity (up to +90%) after vaccination as did peptide No. 73, while control serum reactivity to these peptides was low (Fig 1). Therefore, these peptides were chosen to be further investigated. To prove that serum-reactivity of selected peptides is method-independent binding specificity was re-confirmed using Surface Plasmon Resonance (data not shown).

### Prediction of potential discontinuous stalk epitopes

**Fig 2:**
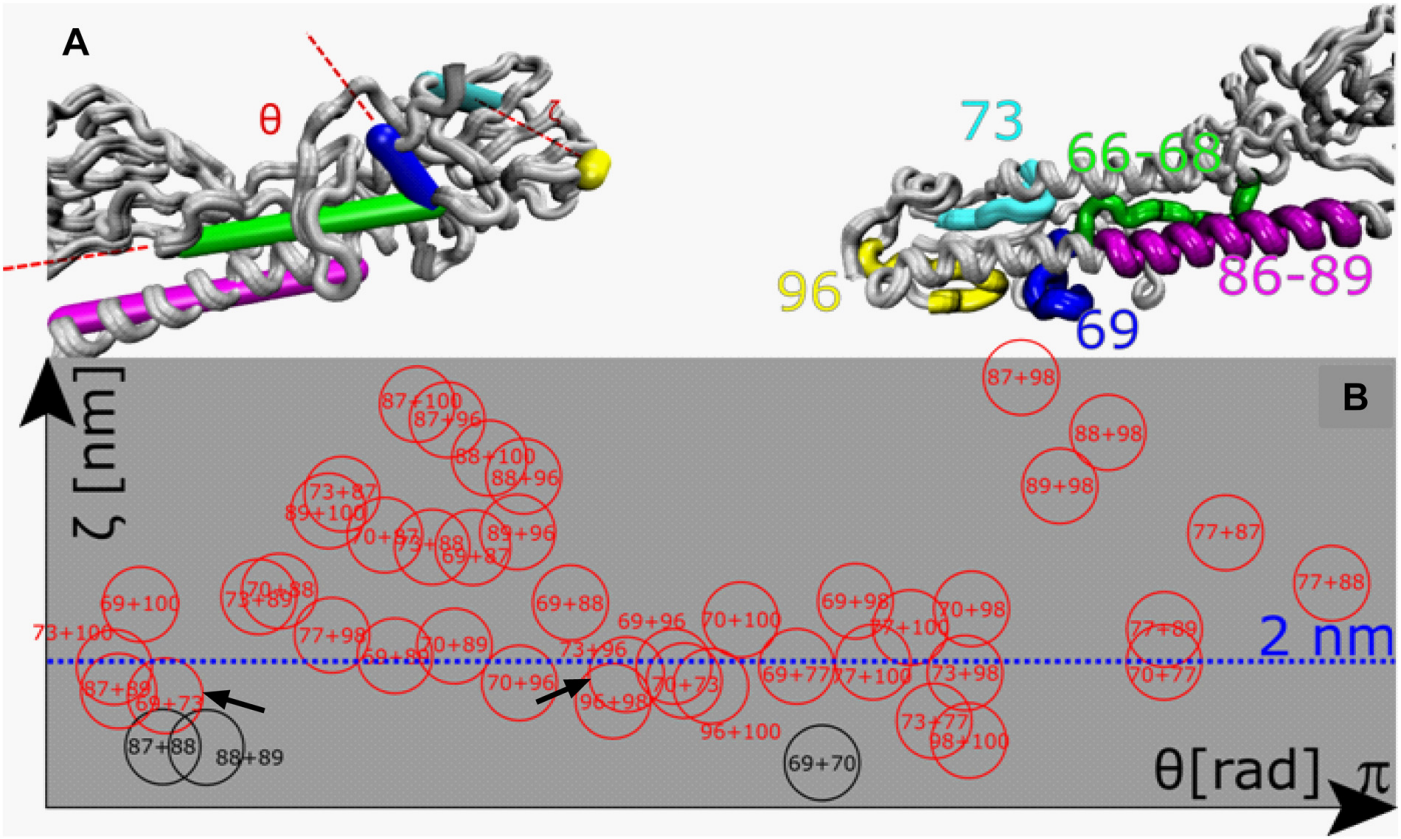
Prediction of potential discontinuous stalk epitopes. The spatial distribution of HA stalk-derived peptides and their relative orientations were assessed with a homologous model of the NC99 HA based on the available HA0 structure of the HA from PR8 (PDB#: 1RU7). The orientation of potential linear epitopes 66–68 (green), 86–89 (pink) and peptides 69 (blue), 73 (cyan) and 96 (yellow) that are part of potential discontinuous epitopes are given as sticks (A, left) or are highlighted in the respective colours in the protein structure (A, right). As peptide 69 is part of the potential linear epitope 66–69 and might also be part of the discontinuous epitope 69+73, it is indicated as separate epitope in the crystal structure. (B) Distances and angles between selected peptide pairs (circles) were calculated to evaluate whether their spatial distribution allows for the formation of a putative discontinuous epitope. Relative distances (abscissa) and relative orientation (ordinate) of HA stalk peptides were computed and peptide pairs as close as < 2 nm were considered suitable as single binding sites of a discontinuous epitope [24] (Black circles) peptide pairs formed by consecutive peptides; (red circles) non-linear peptide pairs. Distances exceeding 3.5 nm are not illustrated.

Besides linear epitopes we were also interested in putative discontinuous/conformational stalk epitopes. The calculation of angles and distances between epitopes served to identify peptides with sufficient proximity in the HA 3D structure to form a single binding footprint of a discontinuous epitope. We used a cut-off of 20 Å, as the footprint of an antibody paratope was shown to encompass a maximum area of approximately 20x30 Å^2^ [24]. Apart from mimotopes [25] linear peptides usually poorly mimic the conformation of discontinuous epitopes in the native protein. Nevertheless, discontinuous epitopes are composed of short linear stretches of 4–7 amino acid residues that form the binding site of an antibody [26]. We assumed that antibodies have only very low binding affinity to one of those single 'stretches' of a discontinuous epitope and would be poorly reactive in the epitope mapping assay. We have therefore also considered peptides with lower antigenicity in the binding assay (Fig 1) and included peptide pairs 69+73 and 73+96 in our experiments (indicated by arrows in Fig 2B). The crystallographic structure of an H1 HA from the closely-related influenza virus A/Puerto Rico/8/1934 (PR8) served to illustrate the native location of the chosen putative epitopes and revealed that peptide Nos. 73 and 96 are located closest to the virus envelope, peptide stretch 66–69 spans the HA1-HA2 intersubunit region including the fusion peptide, whereas peptide stretch 86–89 is located on the long alpha helix (LAH) (Fig 2A). The location of peptide stretch 106–109 could not be visualized as there was no structure available for this part of the HA. Bioinformatic analysis revealed that this stretch covers the HA transmembrane domain.

### Design of an HA protein-based display platform for heterosubtypic HA stalk peptides

We aimed to test, whether antigenic peptides from the HA stalk region contain sufficient antigenic information to exert a protective effect on mice in an *in vivo* challenge experiment. The influenza HA has already been successfully employed as display platform for heterologous epitopes in pre-clinical trials. Epitopes of up to 20 aa in size were engineered into the antigenic site B loop of the HA head domain [18,27–29].

**Fig 3:**
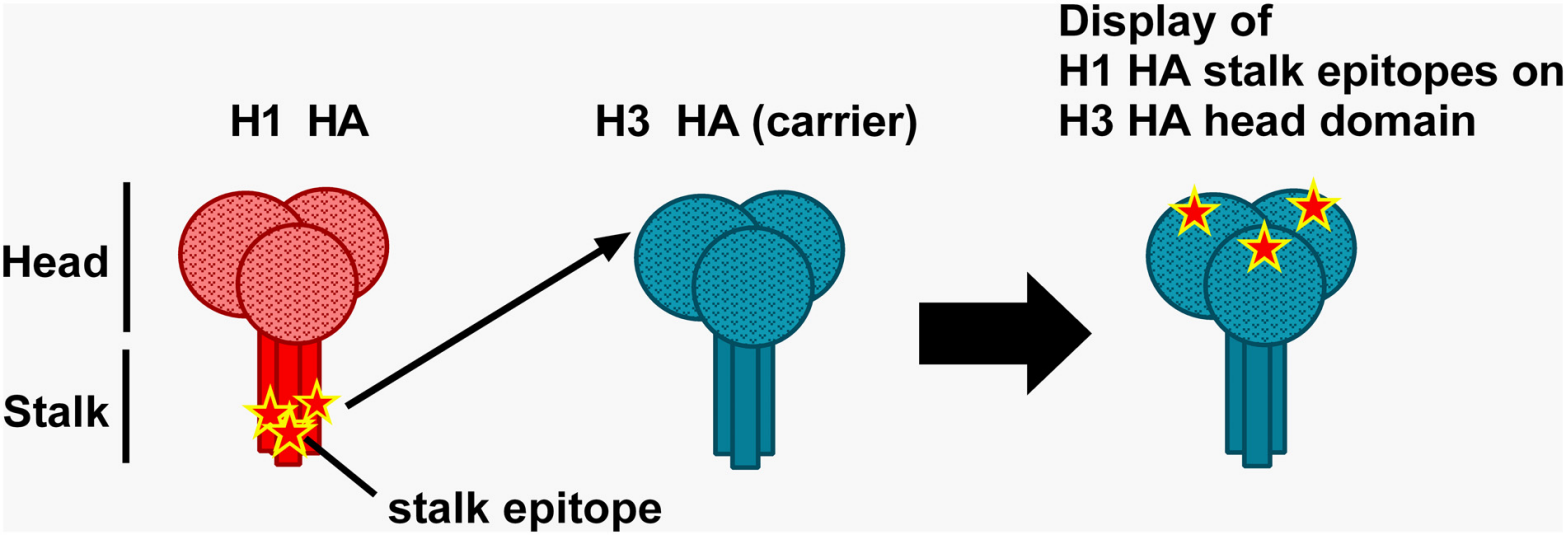
Schematic of the recombinant HA-based display platform for HA stalk peptides. To improve immunogenicity of an identified H1 subtype HA (red) stalk epitope (indicated by a star) we genetically engineered it into the head domain of a heterologous H3 subtype HA carrier protein (blue) to yield a recombinant protein-based display platform for conserved HA stalk epitopes.

**Fig 4:**
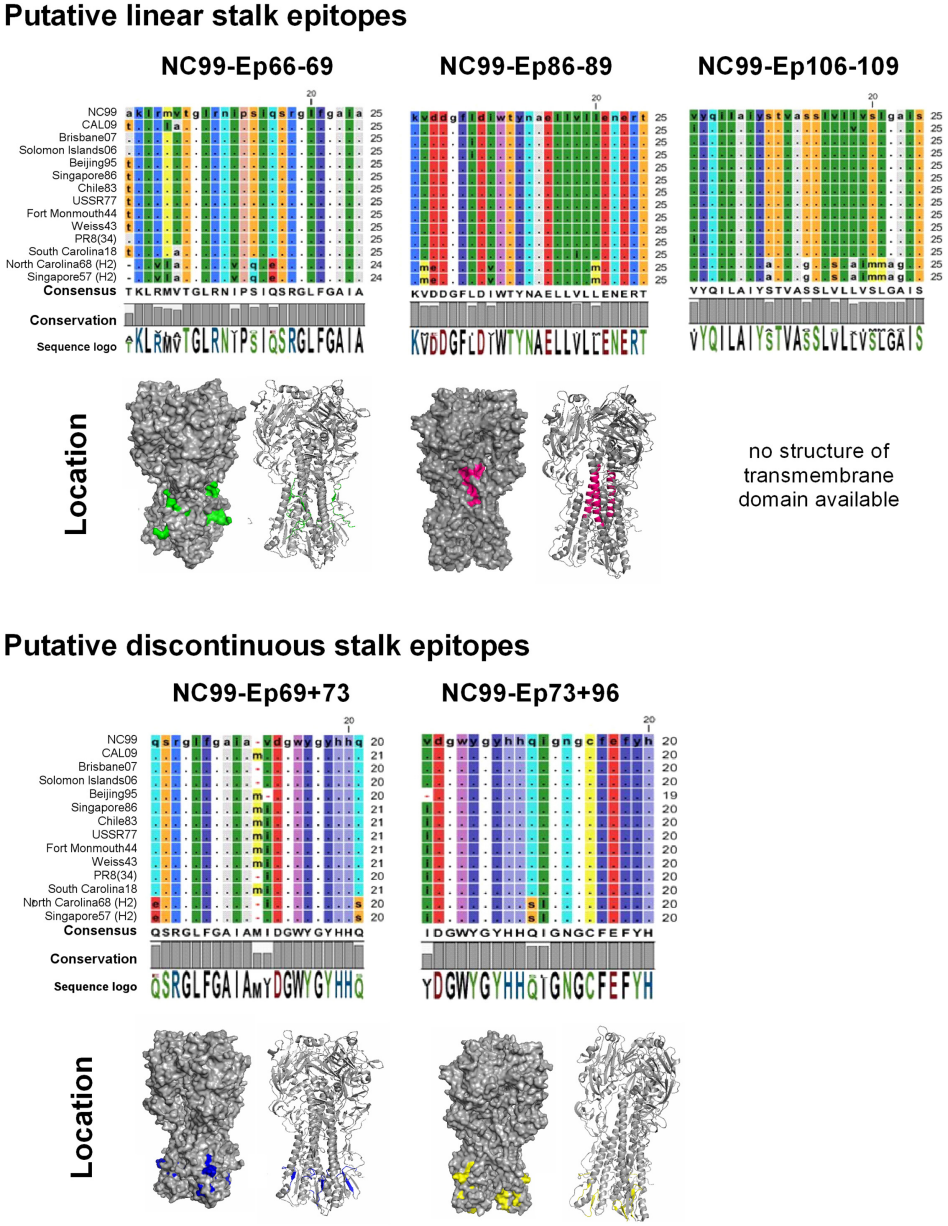
Stalk peptides exhibit a high degree of homosubtypic sequence conservation. Selected peptide sequences from the NC99 HA were aligned to the respective sequences from historical vaccine strains as well as to a pandemic and seasonal representative of the closest phylogenetic HA subtype H2 using CLC Workbench. RasMol colouring of the peptide residues allows to easily identify sequence deviations from the reference sequence from NC99. The consensus sequence is an artificial sequence and reflects the most common sequence in the alignment. The degree of conservation of each residue in the alignment is given by the height of the bars below the consensus sequence (100% conservation is reflected by a full-height bar). The sequence logo displays the frequency/conservation of the residues at each position in the alignment, represented by the relative height of the letters. There, residues are coloured according to their polarity either in green (neutral, polar), red (acidic, polar), blue (basic, polar) or black (neutral, nonpolar). Based on the trimeric structure of the homosubtypic PR8 HA (PDB#: 1RU7), the locations of selected NC99 HA stalk were visualized using PyMol.

We used an H3 subtype HA (phylogenetic group 2) as carrier protein for the identified H1 subtype-derived stalk peptides (phylogenetic group 1) (Fig 3) and decided not to exceed the verified insertion capacity of the B loop by much more than 5 amino acids. As broadly-neutralizing HA-specific antibodies show a neutralization profile that is largely confined to subtypes from the same phylogenetic group [20], cross-protective immunity from the H3 HA carrier in an *in vivo* challenge experiment with an A(H1N1) challenge virus or background signal in a serological assay with an H1 HA was not expected. Stalk peptides selected for being displayed on HA carrier proteins are given in Fig 4. Peptides No. 86 and 107 are either located next to or within an identified antigenic peptide stretch (peptides 87–89 and 106–109 respectively), but had no apparent antigenicity in the binding assay (Fig 1A and S1A Fig). Not knowing, whether these peptides, nevertheless, could have any advantageous effects on the antigenicity of these stretches, we included them as this did not exceed our pre-defined insertion capacity of approximately 25 aa. All selected peptides show a high degree of homosubtypic conservation spanning H1 viruses with more than 90 years of drift, covering two pandemic strains from 1918 and 2009 and historical H1 vaccine strains (Fig 4). HA head-displayed peptide-stretch 106–109 had to be withdrawn from further experiments due to very low expression yields (S4 Fig).

### Displayed stalk peptides do not recapitulate the epitopes of broadly-reactive anti-stalk antibodies

Broadly-reactive antibodies that target the HA stalk domain are considered promising tools for influenza immunotherapy and vaccines that aim at inducing such antibodies are currently in clinical trials [30,31]. Three anti-stalk mAbs C179 [14], 39.29 [15] and CR9114 [16] with largely overlapping epitopes have been crystallographically resolved and were compared to the native location of the four displayed stalk peptides (S3 Fig). While our identified putative linear epitopes (NC99-Ep66-69 and NC99-Ep86-89) do not overlap with these well characterized epitopes, peptide 73, which is displayed as part of the putative discontinuous epitope NC99-Ep69+73 and NC99-Ep73+96, shares four residues (PR8 HA2 residues: I18, D19, G20, W21) with a peptide stretch common to the footprint of all three bnAbs. In addition to that, we experimentally investigated whether our displayed epitopes recapitulate the epitopes of other isolated anti-stalk bnAbs that have not been crystallized. Insect cells expressing full-length HAs displaying stalk peptides have been stained with pan-H1 mAb 6F12 [4] and group 1-specific mAbs 3N6 [23] and KB2 [5]. Flow cytometry results indicate that proteins are expressed on the surface of insect cells as the H3 HA carrier protein is recognised by pan-H3 mAb 12D1 [22]. However, none of the tested pan-H1 or group-1-specific bnAbs binds to our displayed stalk peptides (Table 1).

**Table 1:** Displayed epitopes do not recapitulate the epitopes of previously described broadly-reactive anti-stalk antibodies.

	6F12	3N6	KB2	12D1	No Abs	2° Ab only
Expressed protein	% positive					
**Cells only (-)**	0.0	0.4	0.1	0.1	0.1	0.1
**HIR05 (-)**	0.0	0.2	12.0	21.6	-	-
**CAL09 (+)**	84.8	81.6	77.4	0.1	-	-
**HIR05/NC99-Ep66-69**	0.0	0.2	0.1	24.5	-	-
**HIR05/NC99-Ep86-89**	0.1	0.2	0.0	24.9	-	-
**HIR05/NC99-Ep69+73**	0.1	0.2	0.1	29.0	-	-
**HIR05/NC99-Ep73+96**	0.0	0.2	0.0	26.5	-	-

Non-infected insect cells or cells infected with recombinant baculovirus for the expression of *wildtype* (HIR05, CAL09) or HA-display vectors were stained with anti-stalk pan-H1 mAb 6F12 [4], group-1-specific mAbs 3N6 [23] or KB2 [5] or pan-H3 mAb 12D1 [22] followed by an Alexa Fluor 488-labelled secondary antibody. The fractions of measured A488-positive cells are indicated.

### Vaccination with displayed stalk peptides partially protects mice from lethal heterologous H1 virus challenge

**Fig 5:**
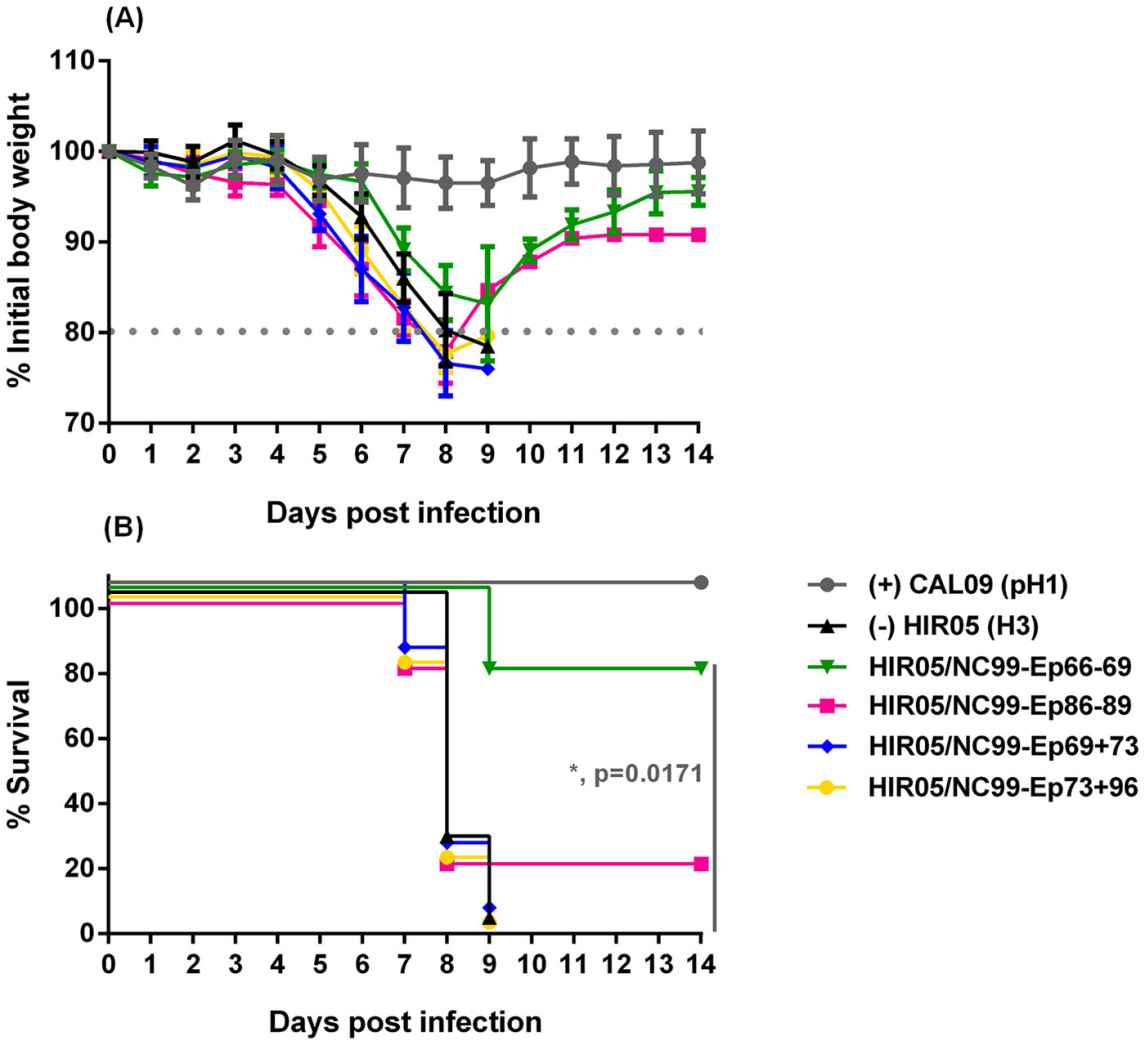
Displayed H1 stalk peptides partially protect mice from lethal heterologous H1 challenge. BALB/c mice (N = 5 per group) received a full-length HA DNA prime (i.m., 40 μg, day 0) and two truncated soluble poly(I.C)-adjuvanted HA protein booster immunizations (i.n. + i.m., 2.5 μg, day 21 and 42) of an H3 subtype HA (HIR05) carrying stalk peptides from an H1 subtype (NC99) virus engineered into its globular head: HIR05/NC99-Ep66-69 (green), HIR05/NC99-Ep86-89 (pink), HIR05/NC99-Ep69+73 (blue) or HIR05/NC99-Ep73+96 (yellow). Control mice received a DNA prime and two soluble adjuvanted protein booster immunizations with *wildtype* H3-subtype HIR05 HA (black) or a single vaccination with the inactivated whole virus vaccine Agriflu^®^ intramuscularly on day 42 (grey). Three weeks later, all mice were intranasally challenged with 5 mLD_50_ of pandemic NL09. Weight loss (A) and survival rates (B) were monitored for 14 days post challenge. The weight loss curves represent the mean percentage of the group initial body weight and error bars indicate the standard deviation. One mouse in the study group HIR05/NC99-Ep66-69 died prior to the challenge experiment and was precluded from the calculations.

As we wanted to assess the ability of displayed NC99-derived H1 HA stalk peptides to confer homosubtypic cross-protection they were tested against a lethal 5 mLD_50_ dose of an unmatched heterologous pandemic NL09 challenge virus. Strikingly, the carrier HA displaying a 25-mer spanning the HA intersubunit region (HIR05/NC99-Ep66-69) was sufficient to protect three out of four mice (75%; p = 0.0171) from mortality in the lethal heterologous challenge experiment (Fig 5B). One mouse in this study group died prior to the challenge experiment and was precluded from further calculations. This group also experienced weight-loss kinetics comparable to the positive control group until day 6 post challenge (Fig 5A). Additionally, one out of five mice (20%; non-significant) immunized with an HA-displayed peptide from the long alpha-helix (HIR05/NC99-Ep86-89) was protected from the lethal challenge. Mice immunized with the two HA-carriers displaying putative discontinuous epitopes (HIR05/NC99-Ep69+73 or HIR05/NC99-Ep73+96) and the negative control group (HIR05) all succumbed to infection between day 7 and day 9 post challenge (Fig 5B). None of the study groups, however, were protected from morbidity, reflected by weight-loss until day 8 or 9 (Fig 5A).

### Displayed H1 HA stalk peptides elicit antibodies that bind the native H1 HA

**Fig 6:**
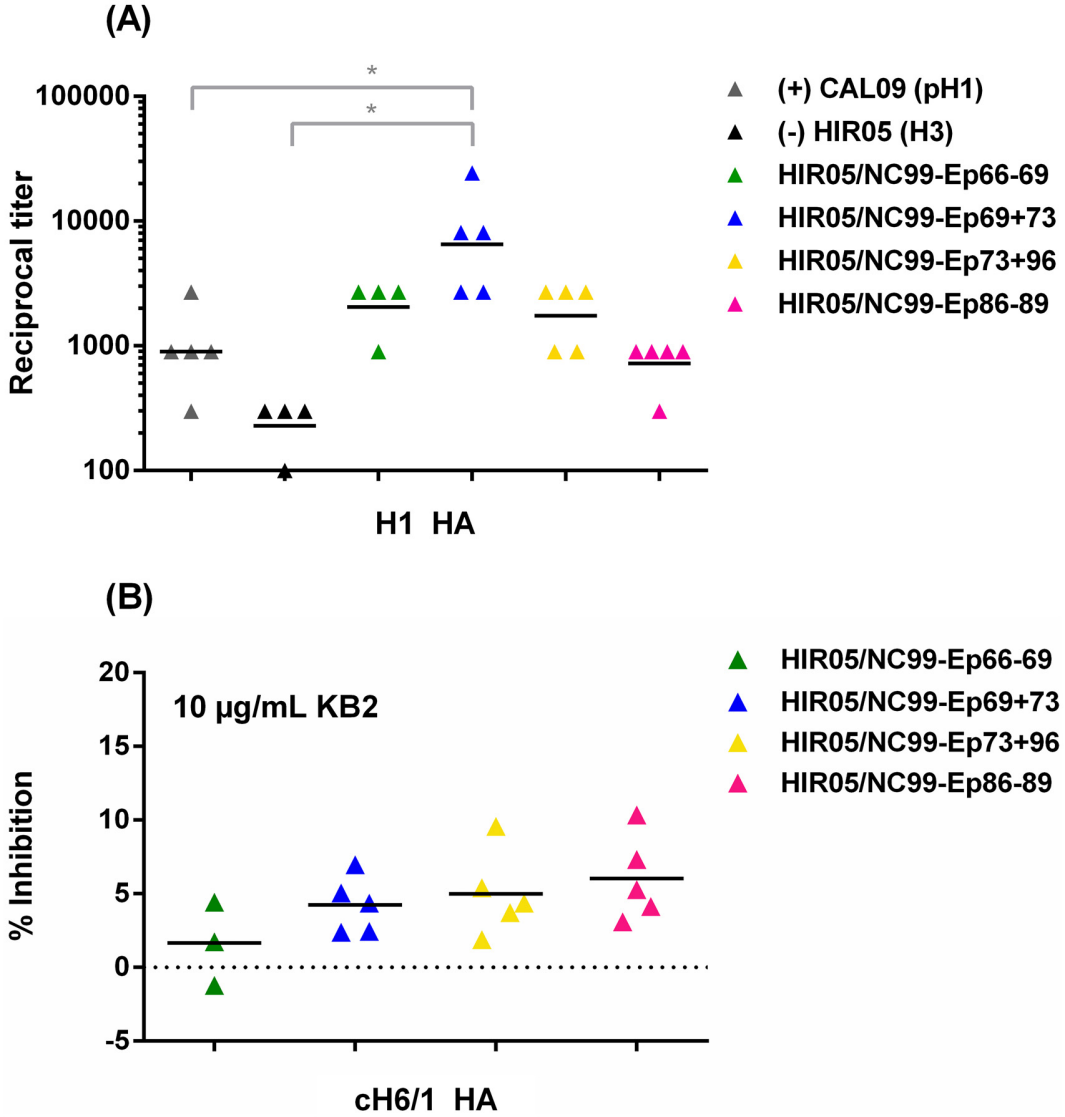
Displayed H1 HA stalk peptides elicit antibodies that bind to the native H1 HA protein but do not strongly compete with anti-stalk mAb KB2. (A) Individual post-vaccination sera were assayed for their reactivity with a recombinant trimeric soluble HA from pandemic CAL09 (H1N1) and (B) were further tested for competition binding to chimeric cH6/1 HA in the presence of 10 μg/mL KB2. Sera were from mice vaccinated with the H3 subtype HIR05 HA (black) or with stalk peptides from a H1 subtype (NC99) virus engineered into its globular head: HIR05/NC99-Ep66-69 (green), HIR05/NC99-Ep86-89 (pink), HIR05/NC99-Ep69+73 (blue) or HIR05/NC99-Ep73+96 (yellow) or from mice vaccinated with the TIV Agriflu^®^ (grey). Due to the lack of enough serum from two mice, there are only three measurements given for vaccine group HIR05/NC99-Ep66-69. * indicates a P value of ≤0.05.

To investigate whether immune sera of mice in our study groups contained antibodies that recognise the parental HA protein, we tested them in a serological assay with soluble trimeric CAL09 HA (containing a different trimerisation domain and purification tag as the HA used for immunization). Generally, we saw a trend of 2-fold higher (non-significant) mean endpoint titers in our vaccine groups HIR05/NC99-Ep66-69 and HIR05/NC99-Ep73+96 as compared to the positive control group (Fig 6A). This suggests that vaccination with the displayed stalk peptides successfully induced stalk peptide-specific antibodies that are able to recognise the parental antigen and that our display system may have improved B-cell recognition of the displayed stalk peptides. The highest mean titer was mounted in the group immunized with a displayed putative discontinuous epitope comprising the fusion peptide (HIR05/NC99-Ep69+73), which was 8-fold higher than in the positive control group (1:1,140 vs. 1:9,180 respectively; p = 0.0393) (Fig 6A). None of the mice in this group, however, were protected from morbidity and mortality (Fig 5).

To further investigate the presence of broadly-neutralizing stalk antibodies in the serum of vaccinated mice we probed serum samples for the presence of KB2-like antibodies as determined by inhibition of the binding of biotinylated KB2 to chimeric cH6/1 HA (having an H6 head on an H1 stalk, [20]) in a competition ELISA. We did not see competitive inhibition of the anti-stalk KB2-like mAb by mouse post-vaccination sera as binding of KB2 was only minimally ablated (up to 10%) in the lowest dilution wells (1:50) (Fig 6B).

## Discussion

Owing to its high degree of sequence conservation and the isolation of stalk-specific antibodies with broad neutralization profiles [16], the immunosubdominant HA stalk has gained increased interest as vaccine target that holds promise in eliciting cross-reactive immunity [2]. Peptide-based vaccines are currently being evaluated in clinical trials [32] and allow the immune system to solely focus on relevant epitopes that are less immunogenic or less exposed in the native antigen. Thus, they provide a potential platform for a stalk-directed vaccine. In the rational design of improved influenza virus vaccines, knowledge about B-cell epitopes is crucial. In addition to X-ray crystallography and site-directed mutagenesis, epitope mapping is a method that is well-appreciated due to its low costs and potential for high-throughput application (as reviewed in Sivalingam and Shepherd [33]).

We performed an epitope mapping experiment with human immune sera from a clinical trial with a monovalent live-attenuated influenza virus vaccine (NC99, H1N1) [3] and synthetic NC99 HA peptides and could show that immunisation with a LAIV can induce antibodies that bind to synthetic peptide targets located both in the HA head and stalk domain (Fig 1A). Mean responses were generally higher with peptides from the head, which might be indicative of the head immunodominance and/or the presence of more linear or micro-conformational epitopes in the head as opposed to the stalk domain. We also observed some degree of seroreactivity in the placebo group (Fig 1B), which may be due to the semi-quantitative nature of the employed assay and due to the fact that measurements were performed in duplicates, which did not allow for the identification of outliers. Previous exposure (and also a different exposure history) to different influenza viruses [34] and host factors such as polymorphisms in human leukocyte antigen, cytokine, cytokine receptor and VH genes [35] may impact the levels of inducible HA-specific antibodies and cause the diversity in the serological profiles we saw among individual sera (S2 and S3 Figs).

Owing to their potential in providing cross-reactive immunity we focused on antigenic peptides from the HA stalk region. We established a soluble recombinant HA-based display platform to enhance immunogenicity of putative stalk epitopes and to evaluate the protective efficacy of selected stalk sequences. For *in vivo* studies, identified epitopes are most commonly conjugated to immunogenic carrier proteins such as the keyhole limpet hemocyanin (KLH), BSA or the tetanus toxoid [36–38]. The manufacturing process of these peptide-vaccines, however, is complex and requires the production of the immunogenic carrier protein, chemical synthesis of peptides and their chemical conjugation to the carriers. This renders the process costly and time-consuming. Moreover, coupling efficiencies to the carrier protein may vary and render the formulation process unpredictable and non-reproducible [39]. Additionally, synthesis and purification of highly hydrophobic peptides may prove challenging or may not work at all, particularly when peptides are poorly soluble in both aqueous and organic solvents [40]. We established a protein-based display platform and engineered identified putative H1 HA stalk epitopes into the immunodominant antigenic site B of an H3 HA head (Fig 3). Epitopes genetically engineered into a protein scaffold demonstrate a defined and highly reproducible entity, as peptides find themselves in the same microenvironment. Moreover, recombinant proteins that display heterologous epitopes can be recombinantly produced at low expenses. Based on the 500 mL expression set-up we used, we could produce between 20 (HIR05/NC99-Ep66-69 and HIR05/NC99-Ep73+96) and 200 (HIR05/NC99-Ep69+73) vaccine doses of 2.5 μg each.

BALB/c mice received a DNA-prime and two adjuvanted protein boosts with our designed immunogens and were challenged with a lethal 5 mLD_50_ dose of a heterologous pandemic H1N1 strain (NL09). We showed that a 25-mer encompassing the intact intersubunit region of the NC99 HA (HIR05/NC99-Ep66-69; covering 18 aa from the HA1 C-terminus and 7 aa from the HA2 N-terminus) was sufficient to protect 75% (p = 0.0171) of the mice from mortality (Fig 5B). This high degree of protection could be seen despite the fact that the challenge virus differed by 3 amino acids within this 25-aa-stretch; the highest degree of genetic variation we see among all our tested peptides (Fig 4). In contrast, Nagy [41] as well as Horváth [42] and colleagues performed challenge studies in mice immunized with 100 μg of an adjuvanted 25-mer peptide with a sequence C-terminally shifted by 5 amino acids (VTGLRNIPSIQSR’GLFGAIAGFIEG) as compared to our tested peptide. A multi-peptide vaccine based on four copies of the HA1 C-terminal part of the intersubunit peptide and a single copy of the HA2 N-terminus protected about 50% of mice from mortality using a lower challenge dose (2 mLD_50_) and a homologous challenge virus [42].

Mice vaccinated with carriers displaying a peptide from the long alpha helix (HIR05/NC99-Ep86-89) were partially protected (20%, non-significant) from mortality (Fig 5B). This region was previously identified by Wang and colleagues to harbour the epitope of 12D1, a pan-H3 neutralizing mAb isolated from mice [22]. In a follow up study they showed that vaccination with two adjuvanted doses of 25 μg KLH-conjugate displaying a 56-mer peptide that encompasses the entire helix resulted in full protection of BALB/c mice in a lethal homologous challenge [38]. We hypothesize that higher antigen doses or the display of a greater portion of the intersubunit region or the LAH would have led to higher protective efficacies of our immunogens. Immunization with HA-carriers displaying putative discontinuous epitopes (HIR05/NC99-Ep69+73 or HIR05/NC99-Ep73+96) did not protect mice from morbidity or mortality (Fig 5A and 5B). This suggests that none of the four peptides (individually or in combination with a peptide that spatially allows for the formation of a discontinuous epitope, Fig 2) carries sufficient antigenic information to be protective in the set-up we tested. Peptide 69 comprises 7 aa of the fusion peptide region (GLFGAIA); the first 9 aa of the HA2 N-terminus (GLFGAIA) form the minimum epitope for 1C9, an anti-H5 HA mAb with cross-clade prophylactic and therapeutic efficacy at doses of 10 mg/kg [43]. We speculate that inclusion of adjacent C-terminal residues would improve protective efficacy of HIR05/NC99-Ep69+73.

We measured relatively low endpoint titers in all vaccine groups, but observed a trend of higher HA-specific antibodies titers in the sera of mice from our vaccine groups than in the control groups (Fig 6A) suggesting that vaccination with our peptide carriers induced antibodies that recognise the parental H1 HA antigen. As our displayed stalk peptides are the only H1 subtype-derived antigenic determinants present within the H3 HA carrier proteins, we assume that H1 HA-specific reactivity in the ELISA stems from stalk-specific antibodies. Highest antibody titers in the vaccine group HIR05/NC99-Ep69+73 do not correlate with their protective efficacy in the challenge experiment (Fig 5). This epitope appears to be well exposed in the stalk of the native HA antigen (Fig 4 and S3 Fig), which may facilitate binding of IgGs in contrast to other tested epitopes. In addition to that, it could be that this displayed epitope better mimics the conformation it would naturally have in the HA protein and therefore induces higher HA-specific antibody titers. We obtained relatively low endpoint titers (1:1,140) in the positive control group (mice immunised once with a TIV). This, however, is in agreement with results obtained by Mullarkey and colleagues in mice, who detected TIV-specific antibodies as low as 1:260 after a single immunisation [44].

Comparison of the location of identified epitopes to the footprints of previously crystallized broadly neutralizing stalk antibodies indicates that our displayed putative discontinuous epitopes (NC99-Ep69+73 and NC99-Ep73+96) share four residues with a peptide stretch common to the epitopes of mAbs C179 [14], 39.29 [15] and CR9114 [16], which have a highly similar footprint involving the HA2 helix A and a few HA1 residues (S3 Fig). Insect cell surface-expressed display vectors were shown not to recapitulate the epitopes of a pan-H1 (6F12) or group-1-specific (3N6, KB2) stalk antibodies (Table 1). The epitope of mAb 6F12 has not yet been elucidated in detail, but it is suggested to share a similar footprint to the aforementioned mAb C179 (S3 Fig) [4], which we have shown to be distinct to our identified epitopes. mAb 3N6 is suggested to bind to the stalk long alpha helix [45] which is the native location of our identified epitope NC99-Ep86-89. As peptide NC99-Ep86-89, however, does not represent the entire long alpha helix (Fig 4) we speculate that the specific LAH segment we have displayed does not accommodate dominant contact residues for 3N6. In a competition assay, sera from vaccinated mice showed negligible competition (up to 10% for HIR05/NC99-Ep73+96 and HIR05/NC99-Ep86-89) with group 1-specific stalk mAb KB2 for chimeric cH6/1 HA (consisting of an H6 head on an H1 stalk) (Fig 6B), suggesting that KB2-like antibodies were not or at very low levels induced by vaccination with our stalk peptide display format. In addition to that, mAb KB2 did not bind to HA-vectored stalk epitopes in the flow cytometry analysis (Table 1). We therefore assume that our displayed epitopes are not overlapping with that of mAb KB2 and that antibodies directed toward our identified epitopes bind distant enough not to sterically hinder binding of KB2.

By virtue of its membrane-proximal location (Fig 4 and S3 Fig) at stretches that undergo serious remodelling during the fusion process [46] we speculate that antibodies binding to NC99-Ep86-89 (located on the HA LAH) could be involved in inhibiting low-pH-induced conformational changes of the proteolytically activated protein. NC99-Ep66-69 spans the fusion peptide region of the uncleaved protein and antibodies induced against this stretch may be involved in preventing HA maturation [47]. As vaccination with the HIR05/NC99-Ep69+73 and HIR05/NC99-Ep73+96 immunogens did not protect mice from mortality nor morbidity (Fig 5), we speculate that induced antibodies (Fig 6A) are non-functional and that the short peptide stretch overlapping with the footprint of bnAbs (S3 Fig) contains too little antigenic information to render these peptides protective. As putative epitopes were identified on basis of their binding characteristics with human immune sera, our assumption was that antibodies were the main mediators of protective immunity elicited by our display-platform. Apart from B-cell epitopes [41,42,48,49], the intersubunit region was also shown to harbour T-cell epitopes [41,42,50,51] and we assume that both arms of adaptive immunity contribute to the protective effect we have seen. It is, however, beyond the scope of this work to elucidate the exact mechanism of protection associated with the identified and displayed stalk peptides, which could also be associated with antibody-dependent cell-mediated cytotoxicity (ADCC) or complement-dependent cytotoxicity (CDC) [52–54].

Nevertheless, in this study we have identified continuous HA stalk sequences that harbour antigenic information relevant for providing protective influenza immunity. These stalk epitopes are distinct to the epitopes of described broadly neutralizing stalk antibodies. We established a novel display platform based on a vaccine antigen that has the potential to be either used as recombinant protein vaccine or platform to evaluate the relevance of viral peptides/epitopes for protection against influenza virus infection. With our display system we could show that 25-mer H1 HA stalk peptides are antigenically sufficient to protect up to 75% of mice from a heterologous H1N1 virus. The HA intersubunit region has proven to be of particular interest as candidate for an influenza peptide-based vaccine, and its full potential has not yet been exploited. Our display format for stalk epitopes induced antibodies that recognise the parental HA antigen. Being used as a prime-vaccine before boosting with conventional seasonal or pandemic vaccines, our stalk epitope carriers could amplify influenza-specific cross-reactive immunity and may be useful for vaccine dose-sparing or for immediate action against emerging strains before conventional vaccines become available.

## Supporting Information

S1 Table Synthesized influenza hemagglutinin (A/New Caledonia/20/1999, H1N1) peptides used in the epitope mapping assay. (DOCX)

S1 Fig Peptide-specific binding pattern of individual human immune sera. Individual responses of human pre-immune and immune sera from twelve individuals receiving a monovalent attenuated A (H1N1) virus vaccine based on NC99 (A) or four placebo recipients (B) are visualized for selected peptides that showed highest mean sero-reactivity with control sera (peptides 8–55, Fig 1) and peptides that were used for the HA-vectored display format (peptides 66–109, highlighted in orange). Dots represent the differences in signal intensity between immune and pre-immune sera of individual donors.
(TIF)

S2 Fig Peptide-specific binding pattern of individual human immune sera in respect to the magnitude of the HI response. Individual responses of human pre-immune and immune sera from twelve individuals receiving a monovalent attenuated A (H1N1) virus vaccine based on NC99 (A) or four placebo recipients (B) are visualized for HA head and stalk peptides with lead sero-reactivity (Fig 1) including stalk peptides that were displayed on the HA carrier. Individual sera were clustered on basis of the magnitude of serum HI response post vaccination and include non-responders, medium-responders and high-responders (no, 2–4 and 16-fold increase in HI activity post vaccination respectively). Dots represent the differences in signal intensity between immune and pre-immune sera of individual donors.
(TIF)

S3 Fig Footprint of crystallized stalk-antibodies in comparison with selected H1 HA stalk epitopes. Identified putative stalk epitopes are compared to the footprint of described crystallized anti-stalk bnAbs C179 [14], 39.29 [15] and CR9114 [16] (red) on basis of the mapped HA structure (PDB#: 1RU7). Arrows indicate a 4-mer stretch of residues common between the identified epitope NC99-Ep73 and the footprint of all three investigated bnAbs.
(TIF)

S4 Fig Chemiluminescent Western blot analysis of insect cell-expressed stalk peptide-carrier Has. Purified insect cell-expressed recombinant soluble HAs were detected using pan-H3 HA-specific mAb 12D1 [22] and developed on X-ray film. Samples were loaded as follows: HAs from H3 subtype HIR05 (lane 1), H1 subtype NC99 (lane 2) and the epitope carrier HAs HIR05/NC99-Ep86-89 (lane 3) HIR05/NC99-Ep106-109 (lane 4) HIR05/NC99-Ep69+73 (lane 5), HIR05/NC99-Ep73+96 (lane 6) and HIR05/NC99-Ep66-69 (lane 7).
(TIF)
