# Supplementary material for: Globular Head-Displayed Conserved Influenza H1 Hemagglutinin Stalk Epitopes Confer Protection against Heterologous H1N1 Virus
Source: PLoS One. 2016 Apr 18;11(4):e0153579. doi: 10.1371/journal.pone.0153579 (PMC4835069; doi:10.1371/journal.pone.0153579)
Supplement: S1 Table — (DOCX) [file pone.0153579.s001.docx]

| **Peptide**  **No.** | **Peptide**  **sequence** | **Peptide**  **No.** | **Peptide**  **sequence** | **Peptide**  **No.** | **Peptide**  **sequence** |
| --- | --- | --- | --- | --- | --- |
| 1 | M-K-A-K-L-L-V-L-L-C | 44 | S-V-V-S-S-H-Y-S-R-R | 87 | F-L-D-I-W-T-Y-N-A-E |
| 2 | L-V-L-L-C-T-F-T-A-T | 45 | H-Y-S-R-R-F-T-P-E-I | 88 | T-Y-N-A-E-L-L-V-L-L |
| 3 | T-F-T-A-T-Y-A-D-T-I | 46 | F-T-P-E-I-A-K-R-P-K | 89 | L-L-V-L-L-E-N-E-R-T |
| 4 | Y-A-D-T-I-C-I-G-Y-H | 47 | A-K-R-P-K-V-R-D-Q-E | 90 | E-N-E-R-T-L-D-F-H-D |
| 5 | C-I-G-Y-H-A-N-N-S-T | 48 | V-R-D-Q-E-G-R-I-N-Y | 91 | L-D-F-H-D-F-N-V-K-N |
| 6 | A-N-N-S-T-D-T-V-D-T | 49 | G-R-I-N-Y-Y-W-T-L-L | 92 | F-N-V-K-N-L-Y-E-K-V |
| 7 | D-T-V-D-T-V-L-E-K-N | 50 | Y-W-T-L-L-E-P-G-D-T | 93 | L-Y-E-K-V-K-S-Q-L-K |
| 8 | V-L-E-K-N-V-T-V-T-H | 51 | E-P-G-D-T-I-I-F-E-A | 94 | K-S-Q-L-K-N-N-A-K-E |
| 9 | V-T-V-T-H-S-V-N-L-L | 52 | I-I-F-E-A-N-G-N-L-I | 95 | N-N-A-K-E-I-G-N-G-C |
| 10 | S-V-N-L-L-E-D-S-H-N | 53 | N-G-N-L-I-A-P-W-Y-A | 96 | I-G-N-G-C-F-E-F-Y-H |
| 11 | E-D-S-H-N-G-K-L-C-L | 54 | A-P-W-Y-A-F-A-L-S-R | 97 | F-E-F-Y-H-K-C-N-N-E |
| 12 | G-K-L-C-L-L-K-G-I-A | 55 | F-A-L-S-R-G-F-G-S-G | 98 | K-C-N-N-E-C-M-E-S-V |
| 13 | L-K-G-I-A-P-L-Q-L-G | 56 | G-F-G-S-G-I-I-T-S-N | 99 | C-M-E-S-V-K-N-G-T-Y |
| 14 | P-L-Q-L-G-N-C-S-V-A | 57 | I-I-T-S-N-A-P-M-D-E | 100 | K-N-G-T-Y-D-Y-P-K-Y |
| 15 | N-C-S-V-A-G-W-I-L-G | 58 | A-P-M-D-E-C-D-A-K-C | 101 | D-Y-P-K-Y-S-E-E-S-K |
| 16 | G-W-I-L-G-N-P-E-C-E | 59 | C-D-A-K-C-Q-T-P-Q-G | 102 | S-E-E-S-K-L-N-R-E-K |
| 17 | N-P-E-C-E-L-L-I-S-K | 60 | Q-T-P-Q-G-A-I-N-S-S | 103 | L-N-R-E-K-I-D-G-V-K |
| 18 | L-L-I-S-K-E-S-W-S-Y | 61 | A-I-N-S-S-L-P-F-Q-N | 104 | I-D-G-V-K-L-E-S-M-G |
| 19 | E-S-W-S-Y-I-V-E-T-P | 62 | L-P-F-Q-N-V-H-P-V-T | 105 | L-E-S-M-G-V-Y-Q-I-L |
| 20 | I-V-E-T-P-N-P-E-N-G | 63 | V-H-P-V-T-I-G-E-C-P | 106 | V-Y-Q-I-L-A-I-Y-S-T |
| 21 | N-P-E-N-G-T-C-Y-P-G | 64 | I-G-E-C-P-K-Y-V-R-S | 107 | A-I-Y-S-T-V-A-S-S-L |
| 22 | T-C-Y-P-G-Y-F-A-D-Y | 65 | K-Y-V-R-S-A-K-L-R-M | 108 | V-A-S-S-L-V-L-L-V-S |
| 23 | Y-F-A-D-Y-E-E-L-R-E | 66 | A-K-L-R-M-V-T-G-L-R | 109 | V-L-L-V-S-L-G-A-I-S |
| 24 | E-E-L-R-E-Q-L-S-S-V | 67 | V-T-G-L-R-N-I-P-S-I | 110 | L-G-A-I-S-F-W-M-C-S |
| 25 | Q-L-S-S-V-S-S-F-E-R | 68 | N-I-P-S-I-Q-S-R-G-L | 111 | F-W-M-C-S-N-G-S-L-Q |
| 26 | S-S-F-E-R-F-E-I-F-P | 69 | Q-S-R-G-L-F-G-A-I-A | 112 | N-G-S-L-Q-C-R-I-C-I |
| 27 | F-E-I-F-P-K-E-S-S-W | 70 | F-G-A-I-A-G-F-I-E-G |  |  |
| 28 | K-E-S-S-W-P-N-H-T-V | 71 | G-F-I-E-G-G-W-T-G-M |  |  |
| 29 | P-N-H-T-V-T-G-V-S-A | 72 | G-W-T-G-M-V-D-G-W-Y |  |  |
| 30 | T-G-V-S-A-S-C-S-H-N | 73 | V-D-G-W-Y-G-Y-H-H-Q |  |  |
| 31 | S-C-S-H-N-G-K-S-S-F | 74 | G-Y-H-H-Q-N-E-Q-G-S |  |  |
| 32 | G-K-S-S-F-Y-R-N-L-L | 75 | N-E-Q-G-S-G-Y-A-A-D |  |  |
| 33 | Y-R-N-L-L-W-L-T-G-K | 76 | G-Y-A-A-D-Q-K-S-T-Q |  |  |
| 34 | W-L-T-G-K-N-G-L-Y-P | 77 | Q-K-S-T-Q-N-A-I-N-G |  |  |
| 35 | N-G-L-Y-P-N-L-S-K-S | 78 | N-A-I-N-G-I-T-N-K-V |  |  |
| 36 | N-L-S-K-S-Y-V-N-N-K | 79 | I-T-N-K-V-N-S-V-I-E |  |  |
| 37 | Y-V-N-N-K-E-K-E-V-L | 80 | N-S-V-I-E-K-M-N-T-Q |  |  |
| 38 | E-K-E-V-L-V-L-W-G-V | 81 | K-M-N-T-Q-F-T-A-V-G |  |  |
| 39 | V-L-W-G-V-H-H-P-P-N | 82 | F-T-A-V-G-K-E-F-N-K |  |  |
| 40 | H-H-P-P-N-I-G-N-Q-R | 83 | K-E-F-N-K-L-E-R-R-M |  |  |
| 41 | I-G-N-Q-R-A-L-Y-H-T | 84 | L-E-R-R-M-E-N-L-N-K |  |  |
| 42 | A-L-Y-H-T-E-N-A-Y-V | 85 | E-N-L-N-K-K-V-D-D-G |  |  |
| 43 | E-N-A-Y-V-S-V-V-S-S | 86 | K-V-D-D-G-F-L-D-I-W |  |  |

**S1 Table. Synthesized influenza hemagglutinin (A/New Caledonia/20/1999, H1N1) peptides used in the epitope mapping assay**
