# Supplementary figures and images for: Globular Head-Displayed Conserved Influenza H1 Hemagglutinin Stalk Epitopes Confer Protection against Heterologous H1N1 Virus
Source: PLoS One. 2016 Apr 18;11(4):e0153579. doi: 10.1371/journal.pone.0153579 (PMC4835069; doi:10.1371/journal.pone.0153579)

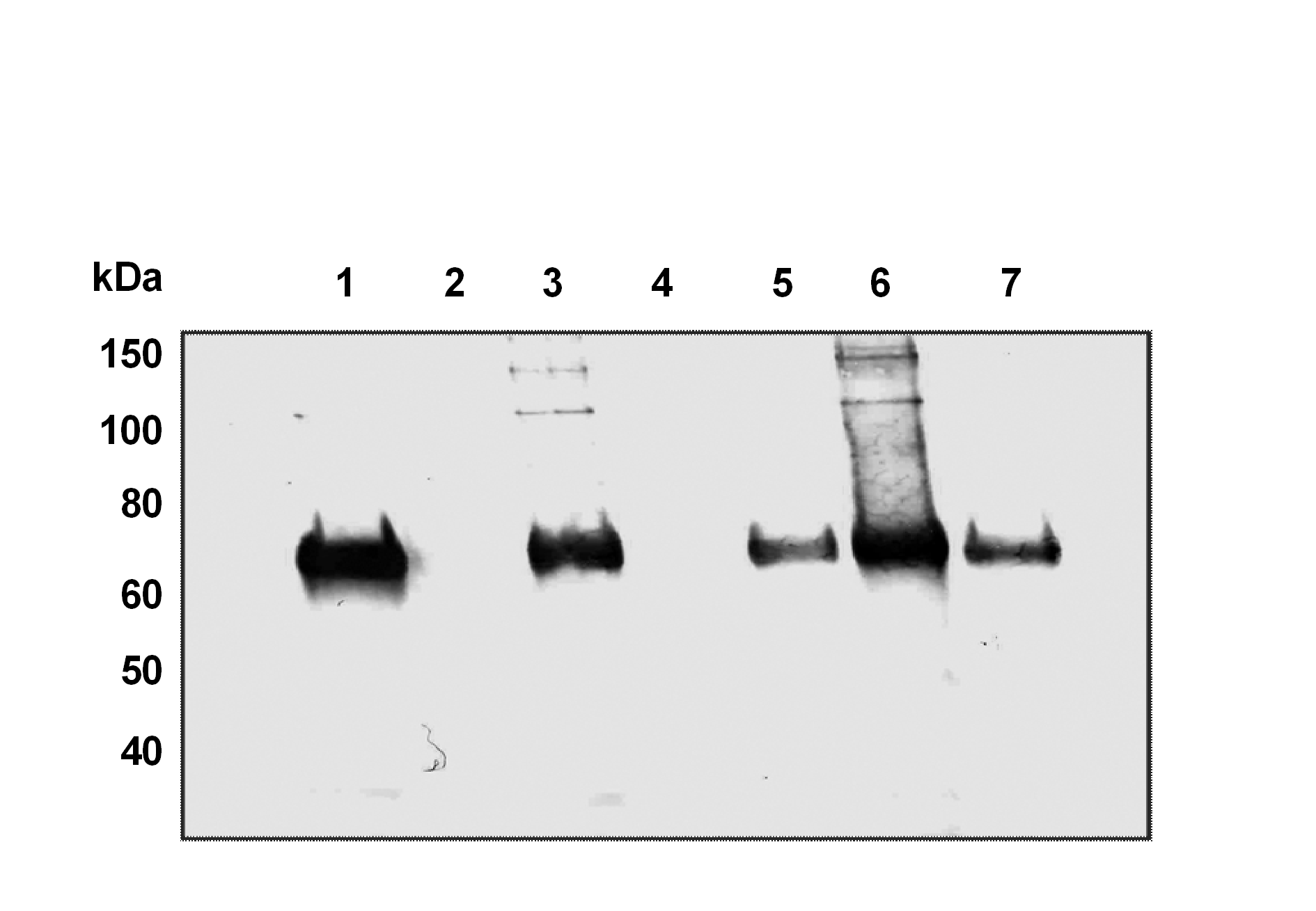

Supplement: S4 Fig — Purified insect cell-expressed recombinant soluble HAs were detected using pan-H3 HA-specific mAb 12D1 [22] and developed on X-ray film. Samples were loaded as follows: HAs from H3 subtype HIR05 (lane 1), H1 subtype NC99 (lane 2) and the epitope carrier HAs HIR05/NC99-Ep86-89 (lane 3) HIR05/NC99-Ep106-109 (lane 4) HIR05/NC99-Ep69+73 (lane 5), HIR05/NC99-Ep73+96 (lane 6) and HIR05/NC99-Ep66-69 (lane 7). (TIF) [file pone.0153579.s005.tif]
